# Electrical Behavior of a Catalyst Composed of Laminar Manganese Oxide Supported on γ-Al_2_O_3_

**DOI:** 10.3390/molecules24162984

**Published:** 2019-08-16

**Authors:** Nayda P. Arias, María E. Becerra, Oscar Giraldo

**Affiliations:** 1 Grupo de Investigación en Procesos Químicos, Catalíticos y Biotecnológicos, Universidad Nacional de Colombia-Sede Manizales, Kilometro 9 vía al aeropuerto, La Nubia, 170003 Manizales, Colombia; 2 Facultad de Ciencias e Ingeniería, Universidad de Boyacá, Carrera 2a Este No. 64–169, 15001 Tunja, Colombia; 3 Laboratorio de Materiales Nanoestructurados y Funcionales, Facultad de Ciencias Exactas y Naturales, Universidad Nacional de Colombia-Sede Manizales, Kilometro 9 vía al aeropuerto, La Nubia, 170003 Manizales, Colombia; 4 Departamento de Ingeniería Química, Facultad de Ingeniería y Arquitectura, Universidad Nacional de Colombia-Sede Manizales, Kilometro 9 vía al aeropuerto, La Nubia, 170003 Manizales, Colombia; 5 Departamento de Física y Química, Facultad de Ciencias Exactas y Naturales, Universidad Nacional de Colombia-Sede Manizales, Kilometro 9 vía al aeropuerto, La Nubia, 170003 Manizales, Colombia; 6 Departamento de Química, Universidad de Caldas, Calle 65 No. 26–10, 17001 Manizales, Colombia

**Keywords:** supported MnO_X_, γ-alumina, ionic conductivity, porous materials

## Abstract

The electrical characterization of catalysts composed of layered manganese oxide in the form of birnessite supported on γ-Al_2_O_3_, which have been successfully used in the combustion of soot, is presented. The results indicate that the electrical conduction and ion conduction processes are influenced by the amount of the active phase. There was also evidence of Grotthuss-type proton conductivity favored by the presence of surface water on the exposed alumina surface. The above is supported by the porous nature of the catalyst in which the surface area varied between 125.2 ± 1.2 and 159.0 ± 1.1 m^2^/g, evidencing changes in the alumina surface. The conductivity, determined from measurements of impedance spectroscopy, at low frequency showed changes associated with the amount of the active phase. The values ranged from 2.61 × 10^−8^ ± 2.1 × 10^−9^ Ω^−1^·cm^−1^ (pure alumina) to 7.33 × 10^−8^ ± 5.9 × 10^−9^ Ω^−1^·cm^−1^, 7.21 × 10^−8^ ± 5.8 × 10^−9^ Ω^−1^·cm^−1^ and 4.51 × 10^−7^ ± 3.6 × 10^−8^ Ω^−1^·cm^−1^ at room temperature for catalysts with nominal active phase contents of 5.0, 10.0 and 20.0%, respectively. Such results indicate that it is possible to modulate the electrical properties with variations in the synthesis parameters.

## 1. Introduction

Heterogeneous metal oxide catalysts are essential in many industrial processes due to their role as mediators in oxidation reactions [1,2], improving the efficiency of those processes. To supply the industrial demand for efficient, selective and highly active catalysts, it is necessary to design synthetic routes allowing the creation of high-performance catalysts. In this sense, the structure, crystal size, morphology and surface area are some of the parameters which influence the selectivity and activity of the catalyst [3]. The properties of these solids can be modulated by changes in the synthesis parameters, among others, supporting the active phase in metal oxides such as Al_2_O_3_, SiO_2_, TiO_2_ or MgO [4]. The most traditional catalysts are made up of noble metals, such as Pd, Pt and Au, due to their catalytic activity, and have been developed using different synthesis methods using the oxides described above as supports [5,6]. However, the disadvantage of their development is their high production costs, which is why they are being replaced by transition metals or by transition metal oxides that are more economical as an active phase [1,4,7]. Among the transition metal oxides, manganese oxides supported in alumina have been used for soot combustion [8], the ozonization of volatile organic compounds [9,10] and the selective catalytic reduction (SCR) of nitric oxide [11], among other reactions, in which a charge transfer is presented between the active center and the reactants. The most common method for supporting manganese oxides is wet impregnation, mainly with acetate salts or manganese nitrate to produce Mn_3_O_4_ or Mn_2_O_3_ as the active phase [9]. Through the thermal reduction of KMnO_4_, previously impregnated in the support, it is possible to produce birnessite materials supported on Al_2_O_3_ [8].

Birnessite is a lamellar phase of manganese oxide that belongs to the family of filomanganates. In this structure, the sheets are formed by octahedral units of MnO_6_ that are joined by the edges and faces in which the manganese is in oxidation states (+4) and (+3), the thickness of the sheets is 4.8 Å and the distance between one sheet and another is approximately 7 Å [12,13]. The heterogeneity of the Mn generates a negative excess charge in the sheets that is compensated by monovalent or divalent cations located in the interlaminar region where water molecules hydrate these cations [13]. The ions in the interlaminar region can be modified by ion exchange reactions, thus modulating the physicochemical properties of birnessite-type manganese oxide. This type of material is a mixed conductor, whose electrical properties come from the transport of intra- and inter-crystalline electrical charges between the agglomerates of particles and the ionic conduction of the interlaminar ions [13,14].

Some studies have suggested that birnessite materials have AC conductivities of 1.60 × 10^−6^ Ω^−1^·cm^−1^ and 6.42 × 10^−5^ Ω^−1^·cm^−1^ at 0.1 Hz and 10 MHz, respectively [14]. It was reported that the electrochemical and electrical properties of these materials also depend on parameters such as their morphology, structure, texture, composition and crystal size [13,14,15]. Combining the semiconducting and ionic conduction nature of birnessite with the high surface area property of γ-alumina, it is possible to improve performance in oxidation reactions [12]. The mechanism of charge transport in supported catalysts is a little-explored aspect of this process. Some studies have reported on the electrical properties of metals, such as Pt and Ni supported in γ-alumina and silica gel, and their relationship with catalytic activity [16,17], as well as charge transport mechanisms in chromium oxide supported in alumina [18]. To the best of our knowledge, there are no studies in which the processes and mechanisms of electrical transport in catalysts formed by supported metal transition oxides are systematically identified. This work explores the interaction of a birnessite-type material with support and concentration effects on the electrical performance of a synthesized material in order to design better catalysts.

## 2. Results and Discussion

### 2.1. Chemical Composition

The chemical composition, obtained by atomic absorption results (AA) (Table 1), shows a variation of the potassium and manganese content of the materials, suggesting that the manganese oxide was successfully supported and is related to the nominal concentration of the precursor used in the synthesis. As previously reported [19], variation in the concentration of the active phase can generate a different interaction with the support.

**Figure 1:**
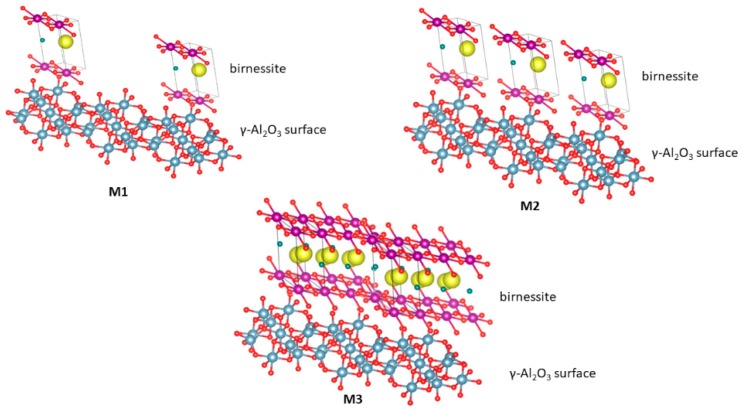
Schematic representation of manganese oxides formed when the content of the manganate ion is increased. M1: 5% by weight of MnO_x_/Al_2_O_3_, M2: 10% by weight of MnO_x_/Al_2_O_3_ and M3: 20% by weight of MnO_x_/Al_2_O_3_. The red circles represent oxygen atoms; the yellow circles represent the K^+^ cations; the violet circle represents the manganese ions; the green circles represent the water molecules; and the blue circles represent Al^3+^ ions.

Figure 1 shows a representation of the possible interactions generated on the surface of the material based on the model of Reed et al. [19]. In M1, the predominant interaction would be between γ-Al_2_O_3_ and manganese oxide (MnO_x_) due to the low concentration of the active phase. For M2, interactions of type γ-Al_2_O_3_-MnO_x_ and MnO_x_-MnO_x_ would be present in the material. In the case of M3, the main contribution would be given by the interaction MnO_x_-MnO_x_ because, at this concentration, the active phase could saturate the support.

### 2.2. Structural Analysis

**Figure 2:**
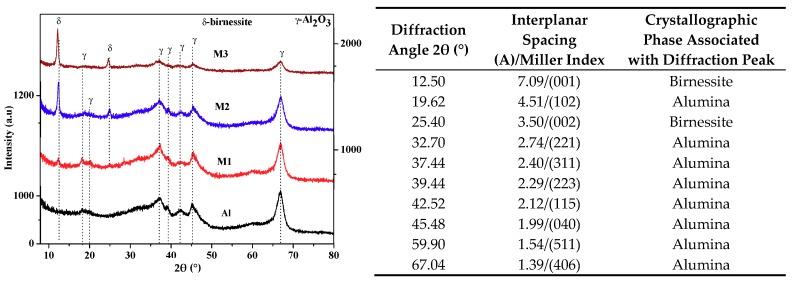
X-ray diffraction patterns of alumina and supported materials. In the table is shown the Miller index of the identified crystallographic phases. The diffractograms show an increase in intensity and a decrease in the width of the diffraction peak associated with birnessite, which suggests an increase in the crystal size of the active phase.

The X-ray diffraction results are presented in Figure 2. The diffraction pattern of the support presents diffraction peaks associated with interplane spacings characteristic of γ-Al_2_O_3_ (JCPDS: 00-046-1215) [19]. In the supported materials, significant changes are observed between 5 and 35° in 2θ (Figure 2, inserted table). The appearance of diffraction peaks of variable intensity in relation to the quantity of the active phase, located at 12.48° in 2θ (7.09 Å) and 25.04° in 2θ (3.55 Å), is consistent with the crystallographic planes (001) and (002) of birnessite manganese oxides [8,12]. Likewise, an increase in the crystal size of the birnessite is present (Table 1) as its concentration in the support increases.

### 2.3. Morphological Analysis and Adsorption Isotherms—N_2_ Desorption at 77 K

**Figure 3:**
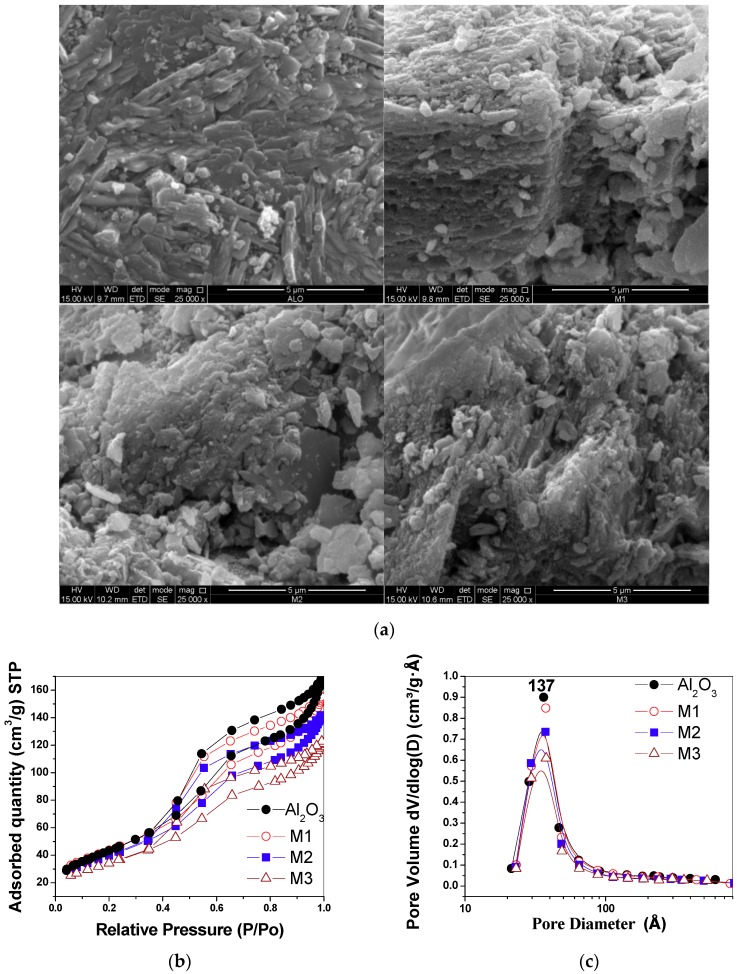
(**a**) SEM micrographs of alumina samples and supported birnessite. Note the rough surface when the manganese oxide is on the support. (**b**) N_2_ adsorption–desorption isotherms. (**c**) Mesopore distribution from the Barret–Joyner–Halenda (BJH) method for the evaluated materials.

SEM was used to study the morphology and surface texture of the synthesized materials. In the γ-alumina micrograph (Figure 3a), small aggregate particles with a smooth-looking surface are observed. In M1, small, compact particles distributed on the surface of the support are seen, giving a rough appearance. In M2 and M3, particles of irregular shape and size and regions exhibiting a porous surface texture are observed. These observations are consistent with the surface modification produced by the interaction of manganese oxide with γ-alumina, as reported previously [8].

In the type IV N_2_ adsorption–desorption isotherms (Figure 3b), according to the International Union of Pure and Applied Chemistry (IUPAC) classification [20], an increase of the N_2_ adsorbed in the supporting materials compared to pure γ-alumina is observed. The hysteresis corresponding to the γ-alumina is of the H4 type, while the support materials present a hysteresis mixture of H3 and H4 types [20]. The variations observed in the hysteresis are related to changes in the shape and accessibility of the pores present in the materials. The pore volume between the materials decreases with an increase in the amount of manganese and with an increase in crystal size (Table 1). This interaction is evident in the monomodal distribution of pore size determined by the BJH method, which presented a peak centered at 37 Å (Figure 3c), indicating a decrease in the adsorption capacity in the mesopores.

### 2.4. Thermogravimetric Analysis

**Figure 4:**
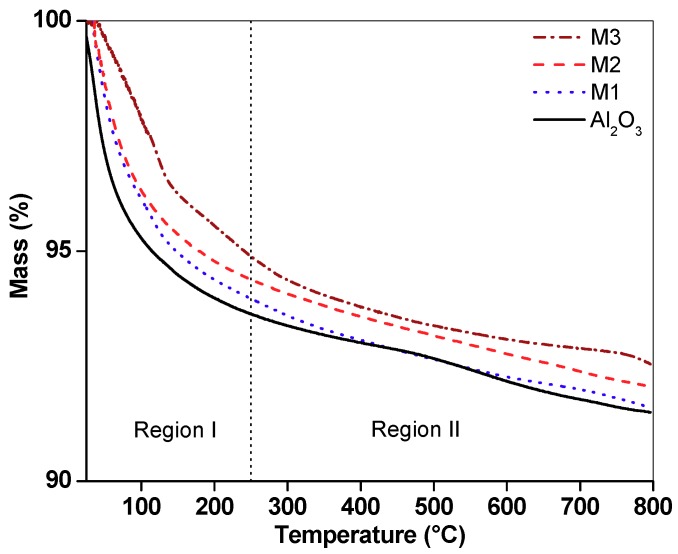
Thermogram for alumina and supported materials M1, M2 and M3 under a nitrogen atmosphere.

TGA curves in a nitrogen atmosphere (Figure 4) show significant thermal events up to 250 °C; the mass losses in this region were higher for alumina (6.35%), followed by M1 (6.04%), M2 (5.62%) and M3 (5.11%). This weight loss was associated with the dehydroxylation of the support surface and loss of water from the interlaminar region of birnessite [12]. Mass losses above 250 °C are attributed to oxygen release due to the transformation of the laminar structure (birnessite) to a tunnel-like structure (cryptomelane) and the crystallization of this phase [8]. Between 250 and 500 °C, the mass losses for the supported birnessite were related to the total content of Mn. The total weight losses were 8.50%, 8.40%, 7.94% and 7.46% for alumina, M1, M2 and M3, respectively.

### 2.5. Nyquist Diagrams and Equivalent Circuits

**Figure 5:**
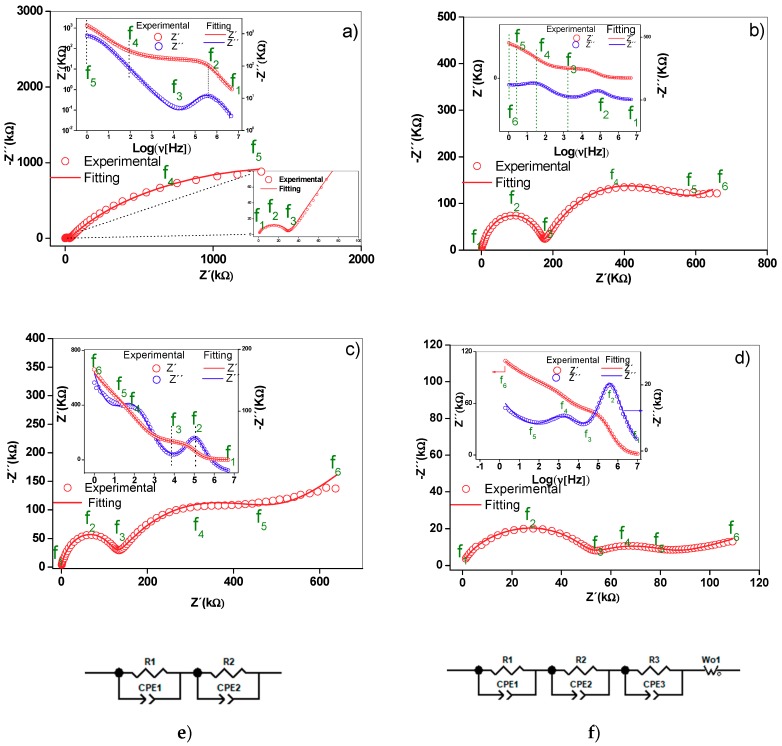

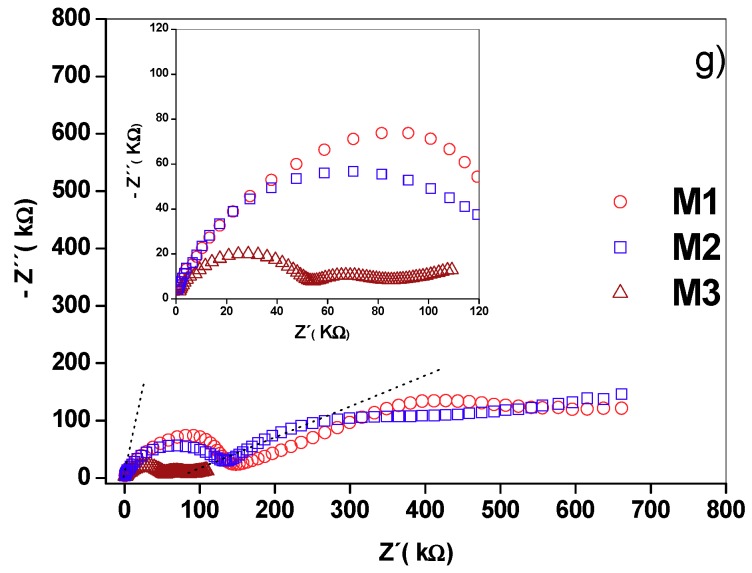
Nyquist diagrams for (**a**) alumina, (**b**) M1, (**c**) M2 and (**d**) M3. Equivalent circuits (**e**,**f**) that have physical meaning and are adjusted to the experimental data. Comparative Nyquist diagram for (**g**) supported birnessite. The Bode diagrams are inserted in the respective Nyquist diagrams.

The Nyquist diagram of the γ-alumina sample (Figure 5a) showed a distorted semicircle and a line at low frequency. In the supported materials (Figure 5b,d), two distorted semicircles were observed centered under the real axis of the impedance, as well as a straight line at low frequencies, the slope of which increased with the active phase concentration. The depression angles of the first semicircle for γ-alumina, M1, M2 and M3 were 17.2°, 11.9°, 14.7° and 21.9°, respectively, while for the second semicircle, they were 32.5°, 47.5° and 53° for M1, M2 and M3. This confirms that non-ideal capacitances are present in the materials [21].

In the materials analyzed, the Al^3+^ ions moving through the vacancies and the superficial protons act as charge carriers in the alumina. In the supported materials, in addition to the species described for the alumina, Al^3+^ contributes to the conductivity charge carriers of the birnessite. Electrons moving by jumps between the Mn^4+^ and Mn^3+^ ions of the MnO_6_ octahedrons, the K^+^ ions found in the interlaminar region and the H_3_O^+^ ions in the surface and the interlaminar region of the birnessite can all contribute to the total conduction [13,14]. Considering that charge carriers have different masses and sizes, they will respond to different frequencies of the electric field. At higher frequencies of the field, the species with higher mass are less affected than those with lower mass. Thus, the electrons and H_3_O^+^ ions would be responsible for the conduction phenomena observed at medium and high frequencies, while in the low-frequency region, the most significant contribution to the conductivity would be the potassium and aluminum ions. The experimental evidence at low frequencies is reflected in a straight line, as shown in Figure 5. As seen in the literature, the presence of this straight line is associated with a Warburg-type phenomenon, which is a generic model of diffusion impedance. Alternatively, this line could be a result of mass transfer occurring due to defects that are part of the structure of the material [21]. In this sense, the ionic conductivity in these materials involves the species found both inside and at the grain boundary.

The two semicircles were assigned to the charge transfer phenomenon in volume (grain interior) and grain boundary at high and medium frequencies, respectively, while the straight line at low frequencies was associated with ionic conductivity [14,15]. The capacitance values estimated from the constant phase elements are presented in Table 2. A difference between 3 and 4 orders of magnitude is observed for the chosen circuit elements.

When assigning the charge transport (electrical) processes to the interior of the grain and at the grain boundary, the order of appearance of the electrical phenomenon was considered. The charge carriers inside the grain will respond faster to the disturbance of the external electric field than those located at the grain boundary. Hence, different relaxation times are presented for each process (Table 3), and therefore the electrical response can be differentiated. Employing a complex dielectric permittivity diagram (Cole–Cole diagram, not shown here), parallel processes associated with protonic conduction were ruled out since a straight line pointing towards low frequencies that have no physical meaning was observed.

**Figure 6:**
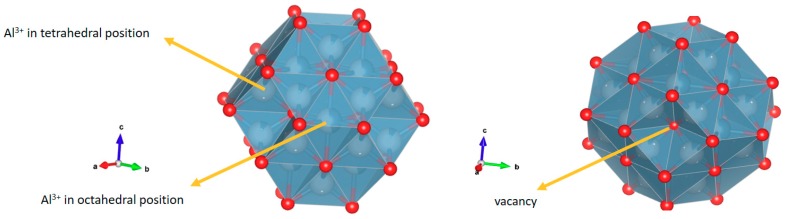
Polyhedral structure of γ-alumina. The red circles represent oxygen atoms, and the white circles represent the K^+^ cations. Al^3+^ atoms are in tetrahedral and octahedral coordination. The polyhedral structure was created in Vesta software based on 9001364 CIF obtained from the Crystallography Open Data Base.

The presence of the two semicircles was assigned to the phenomenon of charge transfer within the grain and grain boundaries at high and medium frequencies, respectively, while the straight line at low frequencies was associated with ionic conductivity. In the structure of the γ-alumina considered as a cubic spinel with vacancies [22,23], Figure 6, with an ideal formula, Al_21+1/3_□_2+2/3_ O_32_ [22], the oxygen atoms are in a cubic packing and the aluminum (3+) ions partially occupy the octahedral and tetrahedral sites to satisfy stoichiometry [22,23].

Thus, the local decompensation of electrical charge in these positions facilitates the transport of charge carriers in the γ-alumina (Figure 5a) involving a transport mechanism by vacancies. It is likely that these charge carriers are Al^3+^ cations, in accordance with structural studies [23]. Additionally, the proton conductivity [24] contributes to the electrical response due to the movement of the charges by means of a Grotthuss mechanism [25,26] favored by the hydroxylated surface of the γ-alumina [26], as indicated by the surface density of molecules (H_2_O/m^2^) calculated by Equation (1).

(1) $$\rho_{s}=\frac{nH_{2}O}{A_{\mathrm{BET}}}$$

where *n*H_2_O corresponds to the number of water molecules estimated from the mass loss up to 250 °C by TGA and A_BET_ is the specific surface area of the material. The estimated surface densities were 1.79 × 10^21^, 1.18 × 10^21^, 1.39 × 10^21^ and 1.37 × 10^21^ molecules of H_2_O/m^2^ for Al_2_O_3_, M1, M2 and M3, respectively. For the supported materials, the contribution to the electrical conduction is attributed mainly to the birnessite and involves an electronic jump mechanism between the octahedra of Mn^4+^ and Mn^3+^, as seen in the birnessite without supporting it. These electrical phenomena occur within the crystal and between particle agglomerates, as reported in previous studies [14,15]. The K^+^ ion located in the interlaminar region of the birnessite contributes to the ionic conduction in the supported materials (Figure 5b–d); however, it is likely that in this conduction process the transport of charge carriers in the interface also involves alumina–birnessite. The Bode diagrams inserted in Figure 5b–d suggest scattering or relaxation processes related to the maximum points of the semicircles present in the Nyquist diagrams. The characteristic frequency of each of the processes is displaced at high frequencies with the increase of the Mn content, indicating greater ease for the transport of charge in M3 associated with the connectivity of the structure due to the MnO_X_-MnO_X_ interactions (Figure 1) on the surface of alumina.

**Figure 7:**
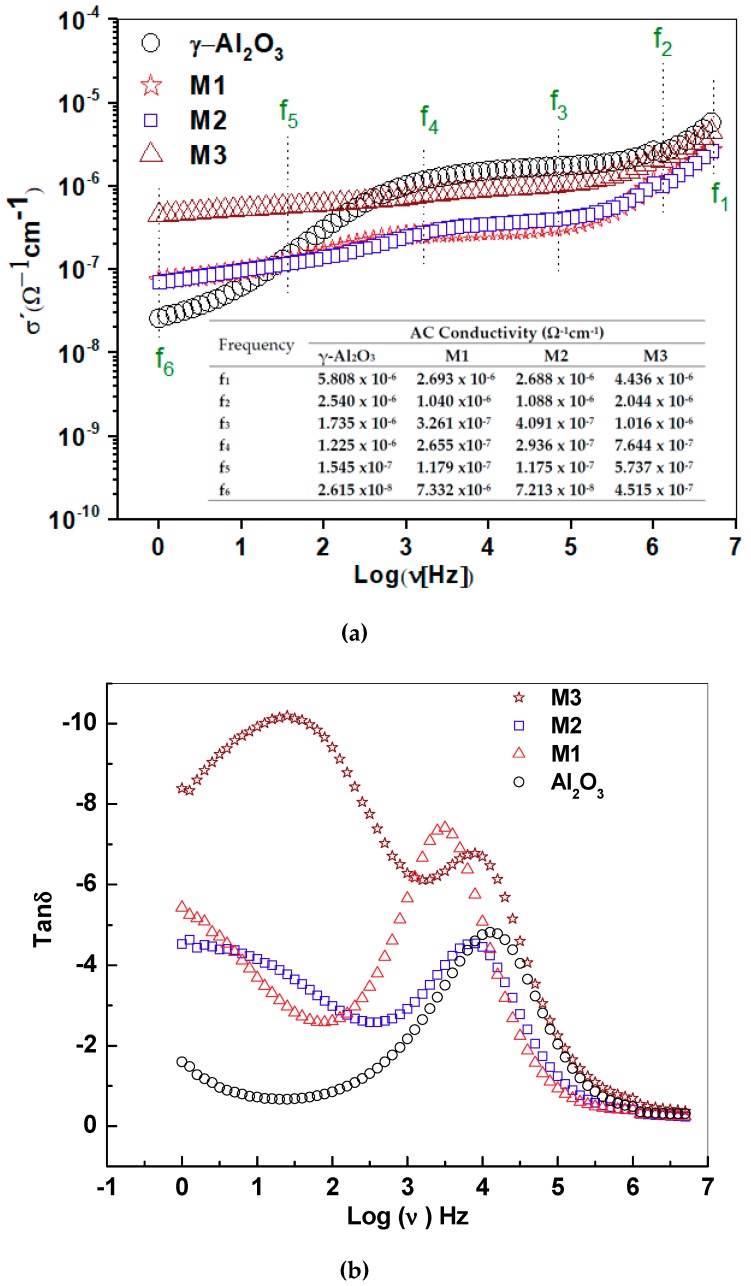
(**a**) Real conductivity is presented as a function of the sample frequency for alumina and the supported birnessites M1, M2 and M3. The table provides values of the conductivity at frequency f_i_. (**b**) Tangent plot of losses (Tan δ) for γ-alumina and supported birnessite. Note that for M3 there is a displacement of two loss peaks, which is associated with the higher content of the birnessite material in the structure.

The experimental results were adjusted with the circuit models shown in Figure 5a–d. In these models, constant phase elements (CPE) were included to consider the effects of the rough surface and the porosity of the materials studied in the capacitance property [15] (Figure 3b). Each circuit element is composed of a parallel pair CPE*i*-R*i*, (*i*: 1, 2, 3) connected in series with a similar pair because the Nyquist diagrams represent series processes [14], and in some cases a Warburg (Wo)-type element was added to represent ionic conduction. Ionic diffusion is a phenomenon of mass transport, manifesting in this work through an electrical property of the material called ionic conduction. Warburg diffusion, as previously indicated, occurs at a low frequency and is represented by a straight line in the Nyquist diagram. There are two methods to evaluate the validity of this element. The first is to adjust the straight-line portion at low frequency and calculate the slope, while the other method is to utilize the data for the Warburg element of the equivalent circuit setting. In this second method, the parameter evaluated is the coefficient Wo1-P. As can be seen in Table 2, the values are lower than the maximum value allowed for this element (0.5), which validates the claim. For γ-alumina, the CPE1–R1 pair reflects the intra-crystalline electrical process (grain interior) while the CPE2–R2 pair was assigned to adjust the low-frequency restricted diffusion data. For the supported materials, CPE3–R3 was added to the circuit model in order to adjust the experimental data considering the transport of charge at the grain boundary. As shown in Figure 5, the proposed circuits adjust the experimental data and differ from that reported by [17] due to differences in experimental conditions such as the type of electrode used and the type of contact between the sample and the electrode. Table 3 presents a summary of the adjusted circuit parameters and shows the changes in the CPE values in each of the charge transport mechanisms. The CPE2 (CPE2-P) coefficient values for each material decrease as the manganese content increases. This observation is consistent with the distancing of the ideal character (Debye type) from the electrical response capacitor due to contact defects between birnessite particles [13,14]. Likewise, the T parameter value relating the effective diffusion thickness with the diffusion coefficient in the Warburg equation (Table 3) increased with the increase in potassium concentration. This coincides with a higher ionic conductivity in M3, as indicated by the low-frequency electrical response in the Nyquist diagram (Figure 5) and the actual conductivity diagram (Figure 7a).

Table 3 shows a summary of the relaxation times at frequencies f_2_ and f_4_. A difference in the magnitude of the relaxation time for M3 (Table 4) was found and is attributed to a higher concentration of manganese on the surface. Therefore, birnessite particles are more interconnected, which facilitates electron mobility.

Table 4 shows the characteristic frequencies for each of the charge transport processes. The characteristic frequency of the process inside the grain (f_2_) is displaced by 84% and 96% towards low frequencies for M2 and M1, respectively. The same behavior in the frequency of M3 was observed at the grain boundary (f_4_), with displacements comparable to the frequency of 60% (M2) and 87% (M1). The characteristic frequency for the initiation of the ionic conduction process (f_5_) was higher in M2 and M3. This suggests that these routes could be obstructed depending on the amount of K^+^ in the interlaminar space and the lower density of water molecules in these materials.

### 2.6. AC Conductivity and Loss Diagram Tan δ

The region of low frequencies in the conductivity spectra is influenced by the DC conduction, as shown in Figure 7a. For γ-Al_2_O_3_, the AC conductivity shows an exponential increase between 1 Hz and 1 kHz and suggests a dispersion behavior at low frequencies produced by the rotation of OH groups on the surface. At frequencies higher than 1 kHz, the plateau suggests DC electrical conduction, possibly due to electric transport between vacant sites. For the supported birnessite, conductivity independence in the frequency between 1 Hz and 1 kHz was observed. Below 2 kHz, the electrical behavior of the supported materials is different from that of γ-alumina, possibly associated with the strong interaction of the electric field with the active phase due to its semiconductor nature. At low frequency, the conductivity is higher for M3, indicating a significant contribution of long-range DC conductivity.

In the region of high frequencies, the conductivity increases in relation to the higher content of Mn that favors the electronic jump between nearest neighbors. The table inserted in Figure 7a shows conductivities at the frequencies of change in the behavior of the real conductivity. The conductivity values for γ-Al_2_O_3_, M1, M2 and M3 at 1 Hz were 2.61 × 10^−8^ ± 2.1 × 10^−9^ Ω^−1^·cm^−1^, 7.33 × 10^−8^ ± 5.9 × 10^−9^ Ω^−1^·cm^−1^, 7.21 × 10^−8^ ± 5.8 × 10^−9^·Ω^−1^ cm^−1^ and 4.51 × 10^−7^ ± 3.6 × 10^−8^ Ω^−1^·cm^−1^, and the conductivities in the grain interior for γ-Al_2_O_3_, M1, M2 and M3 were 1630 × 10^−8^ ± 1.3 × 10^−9^ Ω^−1^·cm^−1^, 1380 × 10^−7^ ± 1.3 × 10^−8^ Ω^−1^·cm^−1^, 520 × 10^−7^ ± 1.2 × 10^−8^ Ω^−1^·cm^−1^ and 1750 × 10^−6^ ± 1.4 × 10^−7^ Ω^−1^·cm^−1^, respectively. These values are consistent with the increase in the content of the birnessite laminar phase on the surface of the alumina.

The literature results for DC conductivity in alumina pellets at room temperature are of the magnitude 10^−10^ [27], while for AC conductivity at 0.1 Hz under a stimulus of 1 V [17], they are 5 × 10^−8^ Ω^−1^·cm^−1^. As such, the alumina values reported in this work are in the range reported in the literature. The Tan δ graph (Figure 7b) shows a symmetric peak for γ-Al_2_O_3_ that suggests a type of dielectric relaxation associated with intrinsic losses in the structure of the material [28], as well as its relationship with the system requirements to change the transport process [13]. Additionally, the frequencies at the Tan δ maximum peaks vary as the amount of birnessite on the γ-alumina surface increases. For the supported materials, these peaks were not symmetric, suggesting a distribution of relaxation times, probably related to the different interactions of the birnessite with the support. These changes were associated with extrinsic losses [29] and are influenced by the pore size distribution. Therefore, for M1, there is a significant energy loss in the process of moving from the interior grain to the grain boundary. This observation agrees with the smaller crystal size, lower manganese content (Table 1) and high surface area (Figure 3b) for this structure. This hinders the continuity in the grain boundary contact due to the high dispersion of manganese oxide on the surface of the support, compared with the other materials. The second substantial variation in energy changes was presented by M3. In M3, the second peak in Tan δ is higher, which suggests that the ion diffusion process becomes more difficult in the interlaminar region due to the higher K^+^ content (Table 1).

### 2.7. Real Permittivity and Dielectric Module

The real dielectric permittivity spectrum (Figure 8a) of the supported materials showed variations at low frequencies with respect to the support, related to the content of Mn and K^+^ in the different materials.

**Figure 8:**
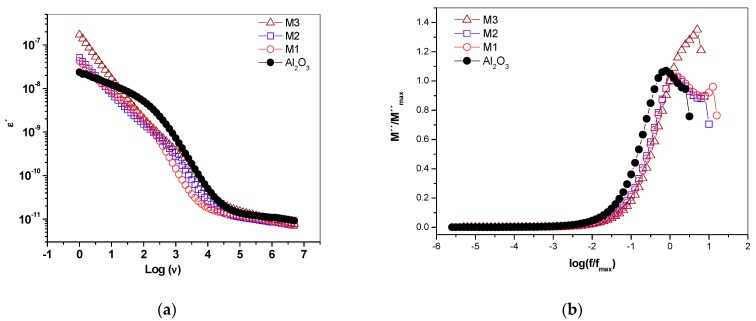
Dielectric spectrum (**a**) real permittivity and (**b**) dielectric module for γ-alumina and supported birnessite materials. For M3, a displacement towards high frequencies occurred at the maximum peak of the dielectric module.

For γ-alumina, a relaxation process around 1 kHz was seen, which becomes less evident as the manganese oxide increases on the support. This relaxation process can be associated with the rotation of water-free molecules on the surface of the material. It is likely that the surface water is found to form “islands” covering the semiconductor surface (Figure 1). Therefore, this suggests that the water–surface interaction is stronger in the birnessite than with the γ-alumina due to the presence of Mn–O–Al bonds in the interface that would modify the hydration energy and the resulting surface water dynamics. Likewise, the water present in the micropores of the semiconductor phase rotates with greater difficulty due to its strong interaction with the walls, as reported by studies on water confined in porous materials [30]. In the dielectric module formalism (Figure 8b), there is a peak indicating a relaxation process moving towards high frequencies as the birnessite content increases. This suggests a relaxation process activated by concentration; after this peak, the driving mechanism is due to short-range electronic hopping [13], as observed in the real conductivity graphs (Figure 7a).

## 3. Materials and Methods

### 3.1. Synthesis

The laminar manganese oxide supported on γ-alumina was synthesized by wet impregnation according to the method reported by Becerra et al. [8]. In a typical synthesis, γ-Al_2_O_3_ (Carlo Erba, chromatographic grade, Val de Reuil, France) was dispersed in different aqueous solutions of KMnO_4_ (J.T. Baker, 99%, Phillipsburg, NJ, USA). The samples were prepared with 5.0, 10.0 or 20.0% by mass of KMnO_4_ as active phase. To obtain 2.00 g of supported material, 100 mL of KMnO_4_ solutions were prepared, ensuring that they contained 0.10, 0.20, and 0.40 g of the KMnO_4_. Subsequently, 1.90, 1.80 or 1.60 g of alumina was added under stirring in order to obtain 5, 10 and 20% KMnO_4_, respectively. The suspensions were heated at 90 °C until the solvent totally evaporated. Subsequently, the materials were calcined at 400 °C for 6 h in an air atmosphere at a heating rate of 10 °C·min^−1^, washed with deionized distilled water (DDW) and dried at 60 °C for 24 h. Samples with 5.0, 10.0 and 20.0% nominal mass of supported KMnO_4_ were named M1, M2, and M3, respectively.

### 3.2. Characterization

Elemental analysis was performed by atomic absorption spectroscopy (AAS) on a Perkin Elmer spectrometer (Perkin Elmer Corporation, Waltham, MA, USA), model 3110. For this purpose, the samples were prepared by fusion with lithium metaborate [8]. The X-ray diffraction patterns of the powder samples were obtained in a RIGAKU MINIFLEX II diffractometer (Rigaku Company, Tokyo Japan), using Cu Kα radiation at 30 kV and 15 mA at a rate of 1° (2θ)·min^−1^, at a size of a step of 0.02° (2θ), between 8° and 80° (2θ). The crystal size was estimated using the Debye–Scherrer equation with the Warren correction [20]. The thermal stability of impregnated materials was studied by thermogravimetric analysis (TGA) in a TA instrument model TGA Q500 (sensitivity: 0.1 μg, resolution: ±0.1 °C) (TA Instrument, Delawere, DE, USA) with a flow rate of N_2_ at 10 mL·min^−1^, at a heating rate of 10 °C·min^−1^. The measurement interval was from room temperature to 800 °C. Differential scanning calorimetry was performed on a DSC Q100 calorimeter (TA Instrument, Delawere, DE, USA) (sensitivity: 0.2 μW, resolution: ±0.05 °C) and the temperature was increased from room temperature to 600 °C at 10 °C·min^−1^.

The adsorption–desorption isotherms of N_2_ used to determine the surface area, pore volume and mesoporous distribution were performed using MICROMERITICS model ASAP 2020 equipment (Micromeritics, Norcross (Atlanta), GA, USA) at 77 K in a range of 1 × 10^−4^ P/Po to 0.99 P/Po for 0.1000 g of sample, which was previously degassed in vacuum at 120 °C for 24 h. The specific surface area was calculated using the Brunauer–Emmett–Teller (BET) method [31]. The Barret–Joyner–Halenda method [31] was used to determine the mesopore distribution pore size. Micrographs were obtained in a FEI Quanta 250 FEG Scanning Electron Microscope (ThermoFisher Scientific, Electron Microscopy Solutions, MSD, Hillsboro, USA) equipped with a secondary electron (SEI) detector operated at 15kV in high-vacuum mode. For impedance spectroscopy analysis, measurements were taken using a dielectric interface SOLARTRON 1296 coupled to SOLARTRON 1260 analyzer with a 12964A sample holder (Solartron Analytical, Farnborough, UK). Data were recorded in a frequency range of 5,0 MHz to 0.1 Hz with a voltage amplitude of 100 mVrms and 0 DC bias. The fitting circuit simulation of AC impedance data was performed with ZView® (Scribner Association, Southern Pines, NC, USA) software.

## 4. Conclusions

The AC conductivity at frequencies higher than 100 Hz for the supported materials suggests short-range conduction, while a low-frequency dispersion occurred in γ-alumina that was related to the rotation of water molecules housed in its surface. This suggests a different interaction of the surface in the supported materials and the alumina in the presence of the electric field.

In the tangent of losses, the maximum energy losses moved towards the low frequency and were related to the concentration of the manganese and potassium in the support. In the same way, the processes of dielectric dispersion were more evident for the alumina than for the supported materials. This indicates that the presence of Mn–O–Al bonds in the interface of the two oxides modifies the hydration energy and the surface water dynamics.

The results suggest proton conductivity in γ-alumina and mixed conduction in supported birnessite-type materials. Therefore, high concentrations of the active phase favor the electronic jump at high frequency due to the continuity in the electric conduction routes influenced by Mn. In this way, the systematic variation of the manganese oxide precursor concentration facilitated the design of a material with higher long-range conductivity (M3) compared to that presented by γ-alumina. It was found that these materials have good catalytic performance in redox reactions.

## Figures and Tables

**Table 1:** Elemental analysis, estimated crystal size, and specific surface area.

Material	K (%)	Mn (%)	Estimated Crystal Size (nm)*	Specific Surface Area (m^2^/g)	Pore Volume (cm^3^/g)
Al_2_O_3_	ND	ND	ND	163 ± 1.1	0.2557
M1	0.73	2.77	3	159 ± 1.1	0.2501
M2	1.80	5.80	4	145 ± 1.0	0.2286
M3	3.59	12.95	11	125 ± 1.2	0.1981

* Estimated crystal size for birnessite phase using the Debye–Scherrer equation. ND: Undetermined.

**Table 2:** Circuital element values that have physical meaning and the adjust experimental results.

Element	Alumina	Error (%)	M1	Error (%)	M2	Error (%)	M3	Error (%)
R2 (Ω)	2.999 × 10^4^	8	1.197 × 10^3^	8	9.010 × 10^2^	8	3.891 × 10^3^	8
CPE1-T (F)	1.099 × 10^−10^	5	3.831 × 10^−12^	6	1.518 × 10^−12^	8	2.092 × 10^−11^	8
CPE1-P	0.86	3	1.18	3	1.28	3	1.03	3
R3(Ω)	3.067 × 10^6^	9	3.623 × 10^5^	8	3.289 × 10^5^	5	2.852 × 10^4^	6
CPE2-T (F)	1.156 × 10^−7^	3	6.927 × 10^−8^	7	6.090 × 10^−8^	9	1.494 × 10^−7^	6
CPE2-P	0.69	5	0.70	3	0.61	5	0.63	4
R4 (Ω)	NA	NA	1.657 × 10^5^	6	1.153 × 10^5^	8	4.335 × 10^4^	4
CPE3-T (F)	NA	NA	4.218 × 10^−11^	2	3.002 × 10^−11^	7	8.136 × 10^−11^	6
CPE3-P	NA	NA	0.91	3	0.93	3	0.89	3
Wo1-R (Ω)	NA	NA	8.323 × 10^2^	8	4.800 × 10^−1^	8	1.017 × 10^3^	8
Wo1-T (s)	NA	NA	4.764 × 10^−7^	7	3.811 × 10^−16^	6	8.401 × 10^−17^	8
Wo1-P	NA	NA	0.21	2	0.19	2	0.02	3

Impedance equation for CPE (constant phase element): $Z=\frac{1}{T{\left(I\times \omega \right)}^{P}}$; Warburg open impedance equation: $Z=\frac{R\times \mathrm{ctgh}{\left[I\times T\times \omega \right]}^{P}}{{\left(I\times T\times \omega \right)}^{P}}$; W1 − T = L^2^/D (L effective diffusion thickness y, D diffusion coefficient); $i=\sqrt{-1}$ y ω = angular frequency of AC signal; NA: not applicable.

**Table 3:** Relaxation times at the maximum points of the semicircles for high and medium frequencies.

Material	Relaxation Time (s)	
	τ_2_	τ_4_
Alumina	4.01 × 10^−7^	6.34 × 10^−2^
M1	2.01 × 10^−6^	5.05 × 10^−5^
M2	1.60 × 10^−6^	1.60 × 10^−5^
M3	7.96 × 10^−8^	6.34 × 10^−4^

**Table 4:** Characteristic frequencies for the Nyquist diagram processes.

Frequency	Alumina	M1	M2	M3
f_1_	5 MHz	5 MHz	5 MHz	5 MHz
f_2_	397.16 kHz	79.24 kHz	99.76 kHz	2.00 MHz
f_3_	19.90 kHz	1.99 kHz	7.92 kHz	50.12 kHz
f_4_	2.51 Hz	31.54 Hz	99.74 Hz	251.12 Hz
f_5_	1.00 Hz	2.51 Hz	19.90 Hz	15.84 Hz
f_6_	N.D	1.00 Hz	1.00 Hz	1.00 Hz

N.D: not detectable.
